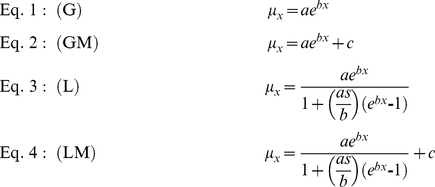# *Wolbachia* Induces Male-Specific Mortality in the Mosquito *Culex pipiens* (LIN Strain)

**DOI:** 10.1371/journal.pone.0030381

**Published:** 2012-03-12

**Authors:** Jason L. Rasgon

**Affiliations:** The Department of Entomology, Center for Infectious Disease Dynamics and Huck Institutes of the Life Sciences, Pennsylvania State University, University Park, Pennsylvania, United States of America; Field Museum of Natural History, United States of America

## Abstract

**Background:**

*Wolbachia* are maternally inherited endosymbionts that infect a diverse range of invertebrates, including insects, arachnids, crustaceans and filarial nematodes. *Wolbachia* are responsible for causing diverse reproductive alterations in their invertebrate hosts that maximize their transmission to the next generation. Evolutionary theory suggests that due to maternal inheritance, *Wolbachia* should evolve toward mutualism in infected females, but strict maternal inheritance means there is no corresponding force to select for *Wolbachia* strains that are mutualistic in males.

**Methodology/Principal findings:**

Using cohort life-table analysis, we demonstrate that in the mosquito *Culex pipiens* (LIN strain), *Wolbachia*-infected females show no fitness costs due to infection. However, *Wolbachia* induces up to a 30% reduction in male lifespan.

**Conclusions/significance:**

These results indicate that the *Wolbachia* infection of the *Culex pipiens* LIN strain is virulent in a sex-specific manner. Under laboratory situations where mosquitoes generally mate at young ages, *Wolbachia* strains that reduce male survival could evolve by drift because increased mortality in older males is not a significant selective force.

## Introduction

*Wolbachia* are maternally inherited α-proteobacterial endosymbionts that infect a diverse range of invertebrates, including insects, arachnids, crustaceans and filarial nematodes. Infection prevalence among taxa is very high; it is estimated that in insects alone, up to 65% of species are infected, making *Wolbachia* perhaps the most wide-spread endosymbiotic bacterium on the planet. *Wolbachia* are responsible for causing diverse reproductive alterations in their invertebrate hosts that maximize their transmission to the next generation. Alterations include parthenogenesis, male-killing, cytoplasmic incompatibility (CI), feminization, and obligate mutualism. These phenotypes have allowed, in many cases, the spread of infection in populations to high frequency [1].

Evolutionary theory predicts that, due to maternal inheritance, *Wolbachia* should evolve in a mutualistic fashion to increase the fitness of infected females [2]. While many studies on the effect of *Wolbachia* infection on insect fitness have been performed, no clear pattern has emerged. *Wolbachia* infections can be mutualistic, parasitic or neutral, or even a combination. For instance, in laboratory studies of the mosquito *Aedes albopictus*, *Wolbachia*-infected females live longer and have more offspring than their uninfected counterparts [3], [4], but these benefits are offset by an increased rate of death in infected larvae [5]. In *Drosophila*, the benefit or cost of *Wolbachia* infection can vary depending on the fly species, the *Wolbachia* strain and is complicated by host effects [6]–[8]. In natural *Drosophila* populations, the evolution of mutualism was observed and was shown to be surprisingly rapid; within 20 years, the *Wolbachia* infection of *D. simulans* in California evolved from a parasite that caused an approximately 20% reduction in fecundity to a mutualistic symbiont that increased fecundity by approximately 10% [9]. The effect of *Wolbachia* on insect fitness is not just of basic scientific interest. Since the daily probability of survival is the most sensitive component in a mosquitoes' role in pathogen transmission, it has been proposed that the invasion of virulent *Wolbachia* strains that shorten vector lifespan could have a significant effect on reducing transmission of vector-borne pathogens. This scenario has been investigated theoretically, where it was shown that under certain conditions, virulent *Wolbachia* strains could spread to high frequency in mosquito populations. This invasion can potentially shift the population age structure toward younger age classes and result in significant reductions in pathogen transmission [10]. Recently, this approach was shown to be potentially feasible by the successful transfer of the virulent *Wolbachia* strain wMelPop into *Aedes aegypti*, where the lifespan of infected mosquitoes was reduced by approximately 50% [11]. CI expression in these transinfected mosquitoes was 100%, and there was no obvious reduction in mosquito fecundity due to infection, suggesting that the virulent infection could invade populations to high frequency [10]–[13].

In the mosquito *Culex pipiens*, *Wolbachia* has been indirectly inferred to enact a fitness cost in some mosquito strains with a genetic background of resistance to organophosphate insecticides (amplified esterase) [14]. Other studies failed to detect positive or negative effect of *Wolbachia* infection on female fecundity under laboratory or field conditions, but the researchers only measured the first oviposition cycle and did not take into account potential effects over the lifespan of the mosquitoes [15], [16]. In this paper, we used cohort life table analyses to investigate the effect of *Wolbachia* on lifetime fitness in the *Culex pipiens* LIN strain. As predicted by theory, no cost of infection was detected in females. However, infection exerted a fitness cost in males, whose lifespan was reduced up to approximately 30% compared to uninfected males. While there are obvious theoretical and empirical advantages for the evolution of mutualism in females, strict maternal inheritance means there is no corresponding force to select for *Wolbachia* strains that are mutualistic in males. Especially in laboratory situations where typically only young mosquitoes mate, *Wolbachia* infections that decrease male fitness could potentially evolve by drift and be maintained.

## Results

**Figure 1:**
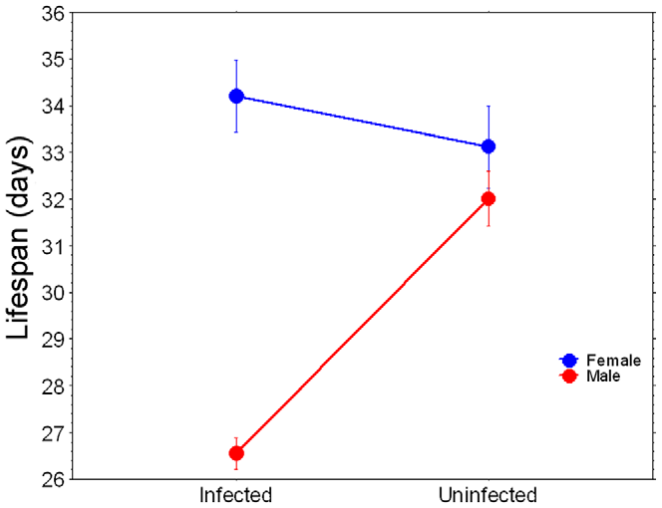
Mean lifespan (days) for mosquitoes in Experiment one. Treatments indicated by different letters are significantly different. Error bars represent standard errors.

**Figure 2:**
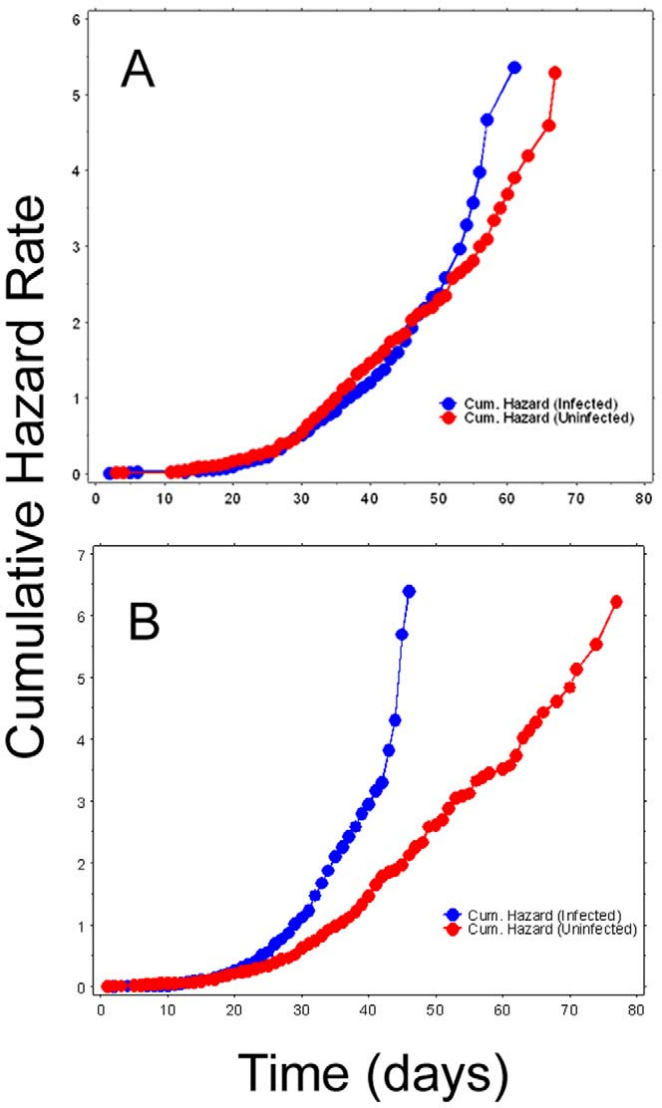
Cumulative hazards rates for mosquitoes in Experiment 1. A) females, B) males.

Non-bloodfed mosquitoes: Infected and uninfected mosquito cage populations were established, but while mosquitoes were allowed access to sugar, they were not bloodfed and an oviposition substrate was not provided. Sex ratios (F/M) were male-biased, but similar between treatments (infected: 0.36, uninfected: 0.39; *P*<0.0001). Due to non-normality of data (Shapiro-Wilks W = 0.985, *P*<0.0001), data were analyzed using non-parametric methods. Kruskal-Wallis test indicated that at least one infection-sex combination differed significantly (tie-adjusted T = 112.9, *P*<0.0001). Pairwise comparisons indicated that while *Wolbachia* infection did not significantly affect the longevity of females, the average lifespan of infected males was reduced approximately 17% compare to uninfected males (Figure 1) (*P*<0.0001). In females, cumulative hazard rates did not significantly differ between infected and uninfected mosquitoes. In contrast, in male mosquitoes cumulative hazard rates begin to diverge after day 20 post-emergence (Figure 2). For all treatments, maximum likelihood simulations executed in WinModest indicated that the logistic model of mortality best fit the observed data for all 4 treatments (Table 1).

**Table 1:** Best-fit model statistics and parameters calculated by WinMoDest.

Experiment	Treatment	LLS	Model	a	b	s	c
Bloodfed	IF	−819.79	L	0.00087	0.15322	1.03630	-
Bloodfed	IM	−2029.18	L	0.00126	0.17939	0.61733	-
Bloodfed	UF	−776.07	L	0.00095	0.16539	1.44211	-
Bloodfed	UM	−1955.45	L	0.00212	0.12803	0.85683	-
Non-bloodfed	IBF	−830.30	G	0.00924	0.06489	-	-
Non-bloodfed	IBM	−770.53	L	0.01042	0.26890	1.07064	-
Non-bloodfed	INBF	−906.42	G	0.00618	0.07784	-	-
Non-bloodfed	INBM	−741.16	G	0.02587	0.10614	-	-
Non-bloodfed	UBF	−580.68	G	0.00854	0.06965	-	-
Non-bloodfed	UBM	−887.04	LM	0.00006	0.68010	2.00000	0.01528
Non-bloodfed	UNBF	−737.03	G	0.00847	0.06112	-	-
Non-bloodfed	UNBM	−863.75	LM	0.00002	0.76203	1.72564	0.02323

I = Infected, U = uninfected, F = female, M = male, LLS = log-likelihood score.

**Figure 3:**
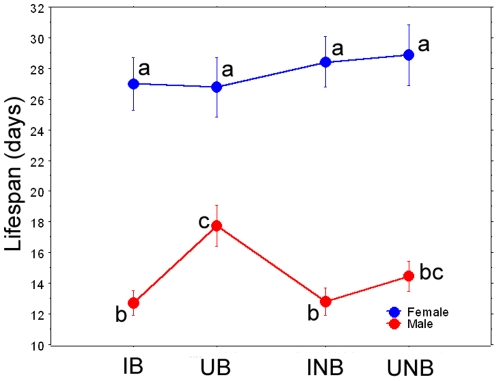
Mean lifespan (days) for mosquitoes in Experiment 2. Treatments indicated by different letters are significantly different. IB = infected, bloodfed, UB = uninfected, bloodfed, INB = infected, non-bloodfed, UNB = uninfected, non-bloodfed. Error bars represent standard errors.

**Figure 4:**
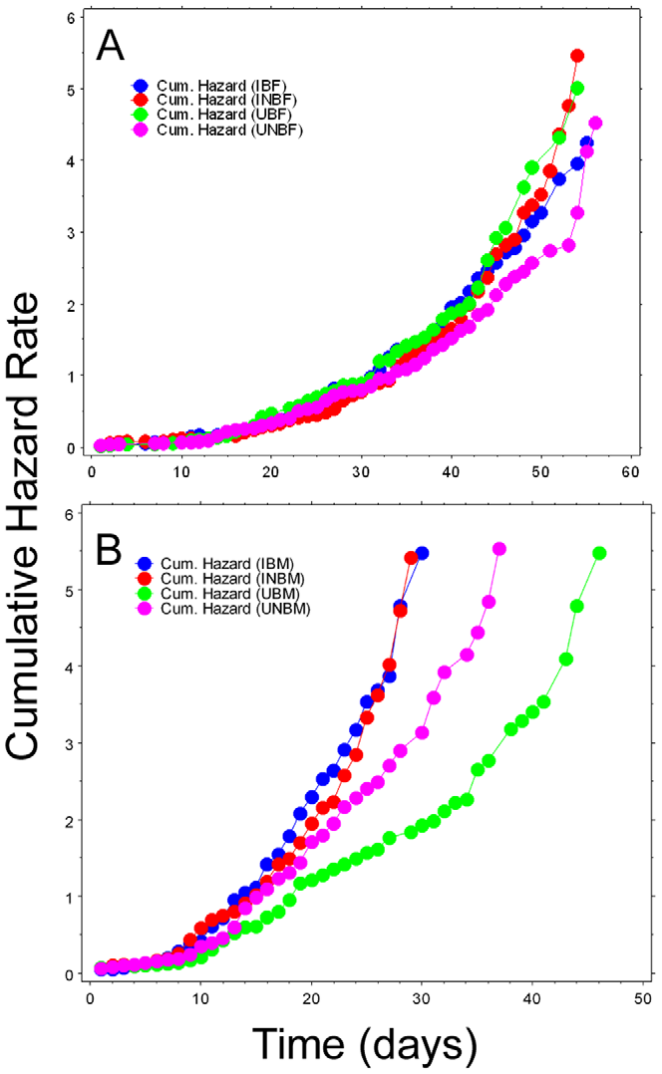
Cumulative hazards rates for mosquitoes in Experiment 2. A: females, B: males. IB = infected, bloodfed, UB = uninfected, bloodfed, INB = infected, non-bloodfed, UNB = uninfected, non-bloodfed.

Effect of bloodfeeding and oviposition substrate: Cage populations were established as described above, but mosquitoes were allowed to feed on a restrained 7-day-old-old chicken once per week and an oviposition substrate (cup filled with strained larval water) was provided at all times for egg depositions. Sex ratios (F/M) in infected treatments (bloodfed and non-bloodfed) did not significantly differ from 1∶1; however, uninfected treatments (bloodfed and non-bloodfed) were significantly male-biased (bloodfed: 0.62, non-bloodfed: 0.72; *P* = 0.01). Due to non-normality of data (Shapiro-Wilks W = 0.956, *P*<0.0001), data were analyzed using non-parametric methods. Kruskal-Wallis test indicated that at least one infection-sex-bloodfeeding combination differed significantly (tie-adjusted T = 533.95, *P*<0.0001). Pairwise comparisons indicated that neither *Wolbachia* infection nor bloodfeeding/reproduction affected the mean longevity of females. However, for males housed with bloodfed females, the average lifespan of infected males was reduced over 28% compared to uninfected males (*P*<0.0001). A similar, but non-significant trend was observed in males housed with non-bloodfed females (11.5% reduction in average lifespan) (Figure 3). Hazard rates for infected males began increasing compared to uninfected males at approximately day 10 for males in the bloodfeeding treatment and day 22 for males in the non-bloodfeeding treatment (Figure 4). Maximum likelihood simulations indicated that the Gompertz model of mortality best fit the observed data for all 4 female treatments, and for infected, non- bloodfed males. The remainder of the male treatments best fit the logistic or logistic-Makeham model (Table 1).

Life-table statistics: Life-table statistics were only calculated for experiment two where mosquitoes were allowed to reproduce. Infected mosquitoes had significantly higher net reproductive rate (*Ro*), higher intrinsic rate of population increase (*r*) and shorter generation time (*T*) compared to uninfected mosquitoes (Table 2), but these observations are possibly not due directly to *Wolbachia* infection. Rather, mosquitoes in the infected cohort began bloodfeeding 2 days earlier than the uninfected cohort, which may or may not be related to *Wolbachia* infection. Nevertheless, the data indicate that there is no obvious fitness cost in females due to *Wolbachia* infection in *Cx. pipiens*.

**Table 2:** Comparison of life-table parameters between infected and uninfected *C. pipiens* mosquitoes.

	Infected	Uninfected
*Ro*	138.9	97.4
*T*	17.3	19.2
*r*	0.284	0.238

## Discussion

Previous studies [15] were not able to detect any fitness costs in *Cx. pipiens* females due to *Wolbachia* infection under laboratory or field conditions. In this study we have confirmed and extended this observation, showing no negative effect of infection on female fitness traits under multiple conditions. However, we did detect, under all conditions examined, reductions in survival in infected males. Calculated life-table statistics do not support any fecundity cost due to *Wolbachia* infection in females – if anything, they suggest the possibility of a slight benefit due to earlier bloodfeeding and oviposition in infected females relative to uninfected females. Due to the cumulative nature of these statistics, even minor events that occur early in life can have major influences on the calculated parameters [17]. Further experiments will be necessary to establish or refute a causal link between *Wolbachia* infection status and mosquito bloodfeeding behavior.

In general, best-fit models supported age-dependent mortality patterns (Logistic or Gompertz) in both infected and uninfected mosquitoes, similar to previous studies [18], [19]. For uninfected mosquitoes allowed access to an oviposition cup, there was an age-independent mortality component as well (Logistic- Makeham) – this is likely due to age-independent drowning of males in this infection treatment. A similar effect was not seen in infected males.

In the laboratory, where mosquitoes reproduce early and generations are not overlapping, *Wolbachia* strains that reduce male survival according to the patterns we observed (no costs in females and late-life induced mortality in males) would have little effect on infection dynamics and could evolve and be maintained by drift. It is likely in the field that males would eventually evolve resistance to the negative effects where these factors may not apply. It remains to be seen how common male-specific virulent *Wolbachia* strains are among laboratory colonies and in nature. Most studies examining fitness effects of *Wolbachia* infection in insects are unlikely to observe this phenomenon since they often focus on females and/or do not examine fitness components using detailed cohort studies. Future studies should examine multiple *Culex* strains, both from established colonies and recently colonized material using cohort life-table analysis.

## Methods

Mosquito strains: Infected mosquitoes used in this study were from the LIN strain, a naturally *Wolbachia*-infected *Cx. pipiens* strain originally collected from Lincoln, CA in August 1999. Uninfected mosquitoes were from the LINT strain, generated from LIN mosquitoes by tetracycline treatment [15]. These 2 strains were genetically similar, as assessed by previous studies [15] and similar low frequency (∼2%) of the recessive sex- linked *crimson* eye color mutation [20].

Rearing: Because mosquito size has been shown to correlate with fitness parameters [21], we reared mosquitoes according to a standardized protocol. 200 first-instar larvae were placed into each 2 L pan with 2.5 g larval food (1∶2∶2 mix of Tetramix fish food, bovine liver powder and ground rabbit pellets. No additional food was added during rearing. Pans were held in a walk-in insectary at 28°C and 80% relative humidity, and randomly rotated on a daily basis to control for insectary microclimate effects. Upon pupation, pupae were placed in experimental cages as described below. Adult cages were held at the same conditions as larval pans.

Survival cages: Cages were custom-built from wood framing and wire mesh screen. Cage dimensions were 1 m×1 m×0.3 m. In all experiments, mosquitoes had access at all times to water and 10% sucrose solution by cotton wicks. Cages were rotated randomly on a daily basis to control for insectary microclimate effects.

Non-bloodfed mosquitoes: In this experiment, mosquitoes were not blood-fed, and an oviposition substrate was not included in the cages. To initiate the experiment, a cup containing approximately 500 24-hour old pupae was placed into each cage. The cup was removed 12 hours later, so that each cage was initiated with similarly-aged mosquitoes. Treatments were *Wolbachia*-infected (LIN) or *Wolbachia* uninfected (LINT) (2 replicate cages per treatment). Cages were checked daily at 12 pm (±0.5 hour) for dead mosquitoes, which were removed from the cage by aspiration and their sex recorded. This continued until all mosquitoes had died.

Effect of bloodfeeding and oviposition substrate: In this experiment, we investigated the effect that mosquito reproduction had on *Wolbachia*-induced fitness effects. Mosquitoes in blood- feeding treatments were allowed to feed once per week on a 1-week old restrained chicken according to approved animal use protocols. To initiate the experiment, a cup containing approximately 500 24-hour old pupae was placed into each cage. The cup was removed 12 hours later, so that each cage was initiated with similarly-aged mosquitoes. An oviposition substrate consisting of a 250 ml plastic cup half-filled with strained larval water was provided in all cages (bloodfed and non- bloodfed) to control for mortality of mosquitoes drowning in the oviposition water. Treatments were (1) *Wolbachia*-infected, bloodfed, (2) *Wolbachia*-infected, non- bloodfed, (3) *Wolbachia*-uninfected, bloodfed and (4) *Wolbachia*-uninfected, non- bloodfed (2 replicate cages per treatment). Cages were checked daily at 12 pm (±0.5 hour) for dead mosquitoes, which were removed from the cage by aspiration and their sex recorded. This continued until all mosquitoes had died.

Statistical analysis: For each experiment, mortality and reproductive data were used to construct treatment and sex-specific cohort life tables [16]. Because data did not conform to parametric assumption, data were analyzed by Kruskal-Wallis test using STATVIEW (SAS Corporation, Cary NC) Pairwise comparisons between infection, sex and feeding treatments were conducted using the Dwass multiple test procedure with a Bonferroni correction for multiple tests using StatsDirect (StatsDirect ltd., Cheshire, UK). Age-specific hazard rates (a measure of instantaneous mortality) were calculated by STATVIEW for all treatments.

To estimate the relative fitness of mosquitoes in experiment 2, we calculated cohort survival (*l_x_*), daily number of female offspring per female assuming a 1∶1 sex ratio (*m_x_*), net reproductive rate (*R_0_ = Σl_x_m_x_*), generation time (*T = Σl_x_m_x_x/R_0_*) and intrinsic rate of increase (*r = ln R_0_/T*) [17]. Net reproductive rate (*R_0_*) gives the total number of eggs a female is expected to lay during her lifetime, generation time (*T*) describes the average age at which females lay their eggs, and intrinsic rate of increase (*r*) describes the instantaneous population growth rate [15]. Because mosquitoes were maintained in group cages, the number of female offspring per female (*m_x_*) is not the exact number of eggs laid by each female, but rather represents the average number of eggs laid on day *x* by the remaining females in each cage on day *x*.

We used WinMoDest [22] to fit the most appropriate mortality model to each individual mortality treatment (infection status, sex, bloodfeeding). The software fits the parameters (a, b, c, s) for a series of semi-hierarchical models (Gompertz [G], Gompertz- Makeham [GM], logistic [L], and logistic-Makeham [LM]) to the data and calculates the log-likelihood for each model. The software allows one to estimate age-dependent [G and L] and age-independent [GM and LM] components of lifespan. Hazard functions for these four models are as follows: